# Revealing the Molecular Regulatory Mechanism of Flavonoid Accumulation in Tender Leaves of Tea Plants by Transcriptomic and Metabolomic Analyses

**DOI:** 10.3390/plants14040625

**Published:** 2025-02-19

**Authors:** Ruiyang Shan, Yongheng Zhang, Xiaomei You, Xiangrui Kong, Yazhen Zhang, Xinlei Li, Lu Wang, Xinchao Wang, Changsong Chen

**Affiliations:** 1 Tea Research Institute, Fujian Academy of Agricultural Science, Fujian Branch of National Center for Tea Improvement, Fuzhou 350013, China; fjnkycys@163.com (R.S.); yxm0593@163.com (X.Y.); zhangyazhen_ada@163.com (Y.Z.); lxlfafu@163.com (X.L.); 2 Key Laboratory of Biology, Genetics and Breeding of Special Economic Animals and Plants, Ministry of Agriculture and Rural Affairs, National Center for Tea Plant Improvement, Tea Research Institute, Chinese Academy of Agricultural Sciences, Hangzhou 310008, China; zhangyongheng@tricaas.com (Y.Z.); wanglu317@tricaas.com (L.W.)

**Keywords:** *Camellia sinensis*, transcriptomics, flavonoids, regulatory network

## Abstract

Flavonoids are secondary metabolites that are beneficial to life activities and are mainly concentrated in buds and leaves in the form of glycosides. Flavonoid glycosides have important effects on the properties and quality of tea plants. Research has shown that the abundance of flavonoid glycosides varies greatly among different cultivars, but research on the regulatory mechanisms that cause their differential accumulation among tea plant cultivars with different leaf colors is lacking. In this study, an integrated analysis of metabolomics and transcriptomics was conducted to determine the regulatory networks regulating astringency and color-related flavonoids in tea plant cultivars with diverse leaf colors. A total of five anthocyanidins, four catechins, and nine flavonol glycosides were found to partially contribute to the differences in taste and leaf color among tea plant cultivars with diverse leaf colors. Furthermore, 15 *MYB* genes and 5 *Dof* genes were identified as potential regulators controlling the expression of eight key structural genes, resulting in differences in the accumulation of specific compounds, including epicatechin (EC), catechin (C), cyanidin, cyanidin 3-O-glucoside, pelargonidin 3-O-glucoside, and quercetin 3-O-glucoside, in tea plant cultivars with diverse leaf colors. These findings provide insights into the development and utilization of resources from tea plants with diverse leaf colors.

## 1. Introduction

Tea has emerged as one of the most significant and widely consumed nonalcoholic beverages globally because of its distinctive flavor profile and inherent nutritional value [1,2]. Tea is roughly divided into six categories based on the processing process, namely white tea, green tea, black tea, yellow tea, dark tea, and oolong tea, and the unique tastes and aromas of these teas are attributed to the differing levels of amino acids, flavonoids, caffeine, and other active compounds present in them [3,4]. Flavonoids are primarily concentrated in buds and young leaves and constitute between 12% and 24% of the dry weight of tea [5]. The principal flavonoid categories include isoflavones, flavanones, flavanols, flavonols, flavones, and anthocyanins [6]. Extensive research has demonstrated that flavonoids possess free radical scavenging and antioxidant properties [5,6,7,8], exerting a profound influence on plant defense mechanisms, growth, and development [9]. Specifically, they influence color formation to attract pollinators, provide UV protection, confer pathogen resistance, mediate plant–microbial signaling interactions, impact pollen fertility, and regulate plant growth [10,11]. Within the human body, the intestinal microflora hydrolyzes glycosylated flavonoids into their respective aglycones, resulting in anti-inflammatory, immunomodulatory, and potent anticancer activities and assisting in the management of cardiovascular diseases [12,13,14].

Furthermore, flavonols and catechins are pivotal flavor constituents in tea liquor, exhibiting dual sensory attributes of bitterness and astringency [15]. Under the catalysis of polyphenol oxidase (PPO), catechins undergo oxidation to form theaflavins, thearubigins, and tea brown pigments, which not only significantly influence the color of tea liquor but also exhibit much lower astringency thresholds compared to catechins [16]. Anthocyanins, a notable subclass of flavonoids, have gained increasing attention due to their roles in cancer treatment and health care [17]. Eight types of anthocyanins have been isolated from purple-leaf tea plant varieties for investigation [18]. These compounds undergo intramolecular coloration via glycosylation and exhibit diverse hues, such as the red appearance of cyanidin-3-O-glucose and cyanidin-3-O-rutin and the dark blue of delphinidin 3,5,3′-O-triglucoside in gentian [19,20]. High concentrations of delphinidin 3-O-(6-O-p-coumaroyl) galactoside and cyanidin 3-O-(6-O-p-coumaroyl) galactoside were detected in ‘Zijuan’ purple-leaf tea in contrast to the minimal levels found in ‘Yunkang 10’ green-leaf tea [21].

Flavonoids accumulate in plant organs like flowers, fruits, and leaves as glycosides, consisting of an aglycone linked to sugar units (e.g., glucose, galactose, and rhamnose). In tea, flavonol glycosides are abundant, accounting for 3–4% of the dry weight, with over 20 aglycones identified [22,23]. These can be categorized as quercetin glycosides, myricetin glycosides, and kaempferol glycosides [23]. Purple-leaf plants are rich in anthocyanins, primarily pelargonidin, cyanidin, delphinidin, peonidin, petunidin, and malvidin [24]. Tea contains anthocyanin glycosides, such as delphinidin and cyanidin derivatives, with β-D-galactoside and β-D-(6-(E)-p-coumaroyl)-galactopyranoside moieties [25,26,27]. All flavonoids originate from the phenylpropanoid pathway, synthesized from L-phenylalanine under the catalysis of a series of enzymes, such as phenylalanine am-monia-lyase (PAL), chalcone synthase (CHS), chalcone isomerase (CHI), flavonoid 3-hydroxylase (F3H), flavonoid 3′-hydroxylase (F3′H), and flavonoid 3′,5′-hydroxylase (F3′5′H), which regulate the early biosynthetic steps of the flavonoid pathway [28,29,30]. Glycosylation is crucial in plants, involving flavonoid UDP-glycosyltransferase (UGT) that transfers sugar moieties to phenolic compounds [22,31,32]. Currently, a series of UGTs have been found to play crucial roles in the glycosylation process of flavonoids in tea plants. For instance, CsUGT73A17 catalyzes the glycosylation of 17 flavonoids specifically at the 7-O position [33], while CsUGT72AM1 is responsible for promoting the glucosylation of kaempferol, quercetin, myricetin, and naringenin, among others, at the 3-O position [18,34]. Additionally, CsUGT73A20 can catalyze the production of various flavonoid glycosides, including flavonoid 3-O-glucosides, flavonoid 7-O-glycosides, flavonoid 3-O-rhamnosides, and flavonoid 3,7-di-O-rhamnosides [22,35]. These studies suggest that UGTs exhibit a complex catalytic role in the glycosylation of flavonoids in tea plants, possessing a diverse range of flavonoid substrates that contribute to the abundant glycosylated flavonoids found in these plants.

In addition to the aforementioned structural genes, transcription factors (TFs) exert crucial regulatory functions in the synthesis of flavonoids. A considerable amount of evidence supports the involvement of MYB TFs in modulating flavonoid synthesis in tea plants. Specifically, CsMYB196 and CsMYB184 exhibit substantial activation of *CsANR* and *CsANS* expression, thereby orchestrating the biosynthesis of catechins, anthocyanins, and flavonols [36]. Similarly, CsMYB34 has been reported to participate in the biosynthesis of galloylated catechins [37]. Notably, CsMYB1 interacts with CsGL3 and CsWD40 to form the MYB-bHLH-WD40 (MBW) transcriptional complex, which subsequently activates the expression of *CsANR* and *CsSCPL1A*, genes involved in galloylated cis-catechin biosynthesis [38]. Furthermore, other TF types, including CsbZIP1 [39], CsWRKY12 [40], CsWRKY48 [41], MYC2 [42], and CsMADSL1 [43], regulate flavonoid synthesis by modulating the transcription of specific flavonoid biosynthetic genes. Interestingly, Dof TFs have demonstrated their capacity to regulate flavonoid synthesis in other plants [44,45]. In tea plants, Dof members are implicated in the regulation of N remobilization [46], chlorophyll metabolism [47], and responses to abiotic stress [48]. However, the role of Dof TFs in regulating flavonoid synthesis within tea plants remains unknown and warrants further elucidation.

A series of tea germplasm resources exhibiting unique leaf colors, distinct from the conventional green color, were observed. These resources are characterized by varying accumulation levels of flavonoids, leading to diversity in tea quality. Recently, numerous studies have focused on the variation in flavonoid metabolism among tea plants with different leaf colors. However, the underlying regulatory mechanisms remain elusive. To address this, our study integrated transcriptome and metabolome analyses to elucidate the potential regulatory network governing specific flavonoid accumulation in tea plants with diverse leaf colors. Special emphasis was placed on the regulatory roles of MYB and DOF transcription factors in flavonoid accumulation in tea plants of different colors. These findings provide crucial foundations for the resource utilization and molecular breeding of tea plants.

## 2. Results

### 2.1. Metabolite Profiles of Leaf Samples from Three Tea Plant Cultivars

**Figure 1:**
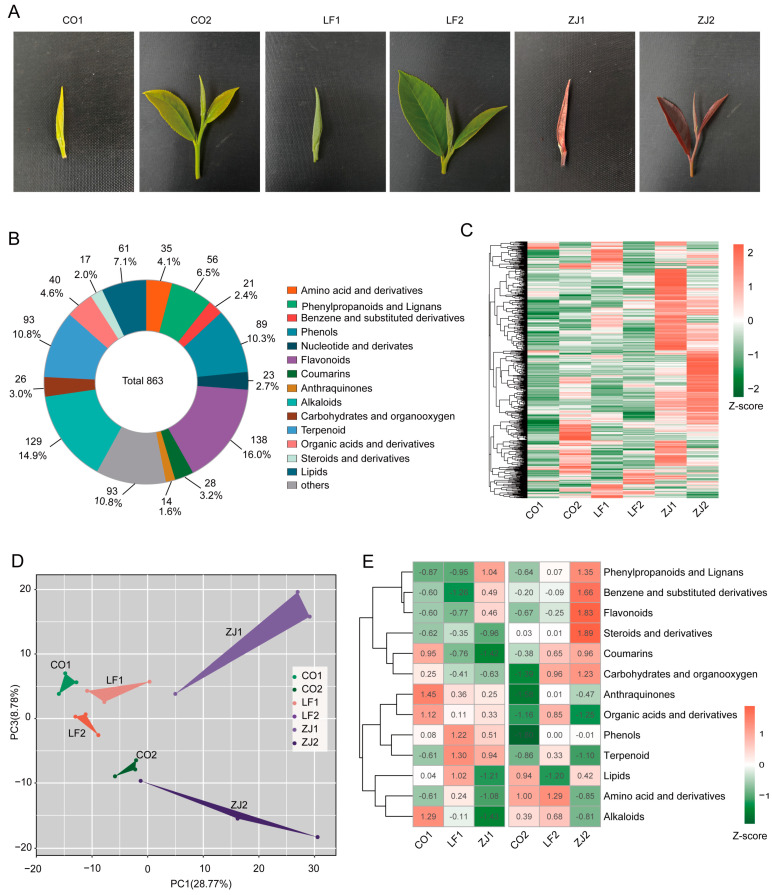
Phenotype and metabolite profiling of tea cultivars with different color phenotypes. (**A**) Leaf phenotypes of CO, LF, and ZJ. (**B**) Classification of total identified metabolites. (**C**) Heatmap displaying accumulation of all metabolites in CO, LF, and ZJ leaves. (**D**) PCA for samples based on all metabolites. (**E**) Heatmap displaying changes in 13 metabolite categories.

By observing the phenotypes of shoots from three tea plant cultivars at different developmental stages, we found that the buds and one bud with two leaves of ‘Mingguan’ (CO) were green and yellowish, whereas those of ‘Lvyafoshou’ (LF) were simply green. In contrast, the buds and one bud with two leaves of ‘Zijuan’ (ZJ) were purple (Figure 1A). A widely targeted metabolite analysis was applied to analyze the differences in their metabolites. A total of 863 components were detected, including 35 amino acids and derivatives, 56 phenylpropanoids and lignans, 21 benzene and derivatives, 89 phenols, 23 nucleotides and derivatives, 138 flavonoids, 28 coumarins, 14 anthraquinones, 129 alkaloids, 26 carbohydrates and organooxygen compounds, 93 terpenoids, 40 organic acids and derivatives, 17 steroids and derivatives, 61 lipids, and 93 others (Figure 1B and Table S2). A heatmap of all 863 components reveals that they had different accumulation patterns (Figure 1C), and the PCA results indicate that the metabolite profiles of the six samples were separated (Figure 1D). A heatmap was generated to assess the total levels of 14 metabolite categories in different leaf samples of CO, LF, and ZJ. The results reveal that the total levels of anthraquinones, organic acids and derivatives, and alkaloids were greater in CO1, whereas LF1 accumulated more phenols, terpenoids, and lipids. The most abundant phenylpropanoids and lignans, benzene and derivatives, flavonoids, steroids and derivatives, and carbohydrates and organooxygen compounds were found in ZJ2, whereas the most abundant amino acids and derivatives were found in LF2 (Figure 1E). These results collectively indicate that the samples of the buds and one bud with two leaves of the three tea plant cultivars have distinct metabolite profiles.

### 2.2. Differentially Abundant Metabolite Analysis

**Figure 2:**
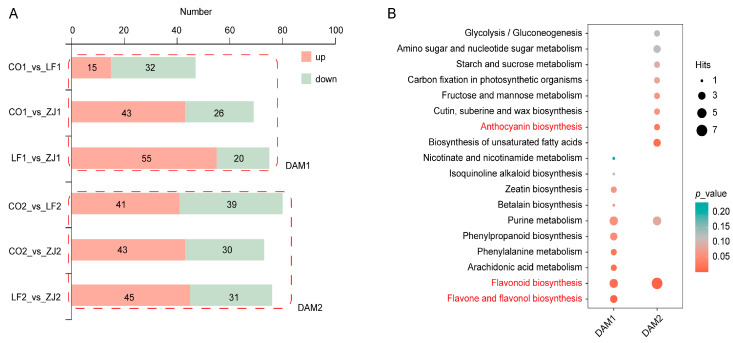
Phenotype and metabolite profiling of tea cultivars with different color phenotypes. (**A**) Leaf phenotypes of CO, LF, and ZJ. (**B**) Classification of total identified metabolites.

Six comparison groups, namely CO1_vs_LF1, CO1_vs_ZJ1, LF1_vs_ZJ1, CO2_vs_LF2, CO2_vs_ZJ2, and LF2_vs_ZJ2, were set to identify significantly differentially accumulated metabolites (DAMs), resulting in 47, 69, 75, 80, 73, and 76 DAMs, respectively (Figure 2A and Table S3). DAM1 (consisting of CO1_vs_LF1, CO1_vs_ZJ1, and LF1_vs_ZJ1) and DAM2 (consisting of CO2_vs_LF2, CO2_vs_ZJ2, and LF2_vs_ZJ2) were subsequently defined to reflect the total DAMs of the samples of buds and one bud with two leaves, respectively. A KEGG enrichment analysis revealed significant differences in the top 10 enriched pathways between DAM1 and DAM2 (Figure 2B), indicating distinct metabolic characteristics of the samples of buds and one bud with two leaves. Notably, ‘flavonoid biosynthesis’ was significantly enriched in both DAM1 and DAM2, with ‘flavone and flavonol biosynthesis’ and ‘anthocyanin biosynthesis’, the different continuations of ‘flavonoid biosynthesis’, enriched in DAM1 and DAM2, respectively (Figure 2B). These results reveal significant differences in the accumulation of flavonoid compounds in the samples of both the buds and one bud with two leaves.

### 2.3. Accumulation Patterns of Anthocyanidins, Flavan-3-Ols, and Flavonol Glycosides

**Figure 3:**
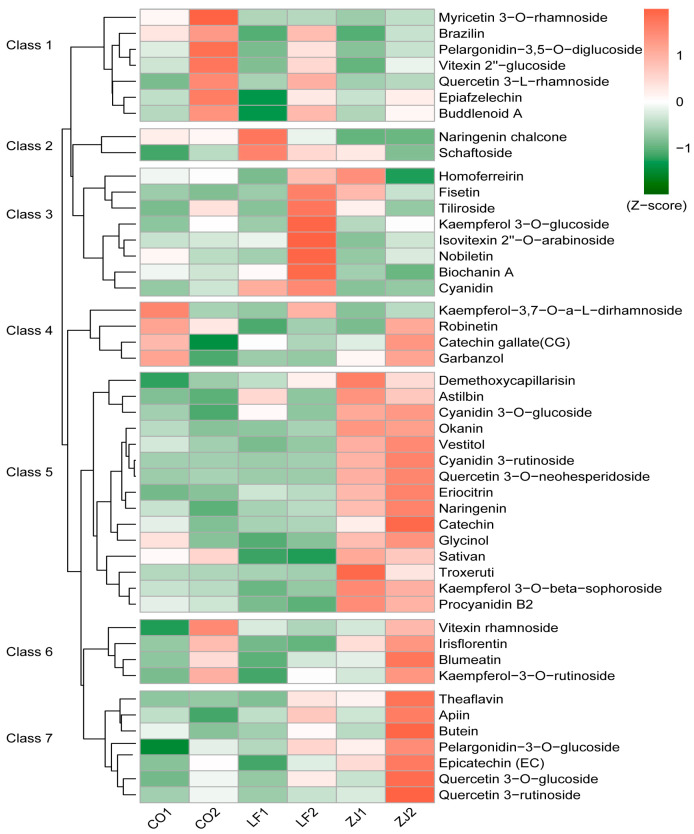
The accumulation profiles of 47 differentially accumulated flavonoids.

A total of 47 differentially accumulated flavonoids were found in all of the compared groups and clustered into seven different accumulation patterns (Figure 3). The compounds clustered into groups 1, 2, 3, and 7 presented the greatest accumulation of CO2, LF1, LF2, and ZJ2, respectively. Cluster 4 compounds, with the exception of kaempferol-3,7-O-alpha-L-dirhamnoside, were more highly accumulated in CO1 and ZJ2. The cluster 5 compounds accumulated more in ZJ1 and ZJ2, whereas the cluster 6 compounds accumulated more in CO2 and ZJ2.

**Figure 4:**
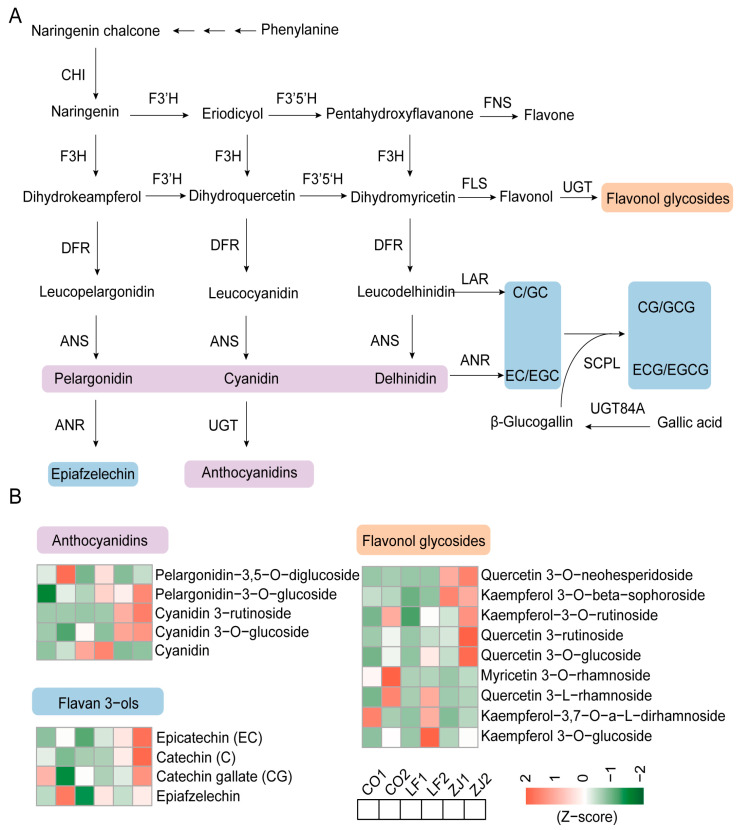
Differentially accumulated flavonoids in CO, LF, and ZJ leaves. (**A**) Biosynthetic pathways of anthocyanidins, flavan 3-ols, and flavonol glycosides. (**B**) Differentially accumulated anthocyanidins, flavan 3-ols, and flavonol glycosides.

Considering that anthocyanidins are important compounds that result in differences in leaf color phenotypes, flavan-3-ols alongside flavonol glycosides are important flavonoids that contribute to the formation of characteristic flavors in tea. Therefore, these metabolites attracted our attention, and their biosynthetic pathways (Figure 4A) and accumulation patterns (Figure 4B) were further investigated. Five differentially accumulated anthocyanidins were detected, among which cyanidin was more highly accumulated in LF1 and LF2, whereas pelargonidin-3,5-O-diglucoside and pelargonidin-3-O-glucoside presented the greatest accumulation in CO2 and ZJ2. Notably, similar to procyanidin B2 (Figure 3), cyanidin 3-rutinoside and cyanidin 3-O-glucoside were significantly more abundant in ZJ1 and ZJ2. Four flavan 3-ols and 9 flavonol glycosides were differentially accumulated among these samples. Three flavan 3-ols (EC, C, and CG) and 3 flavonol glycosides (quercetin 3-rutinoside, quercetin 3-O-glucoside, and kaempferol 3-O-rutinoside) exhibited the greatest accumulation in ZJ2, whereas 1 flavan 3-ol (epiafzelechin) and 2 flavonol glycosides (myricetin 3-O-rhamnoside and quercetin 3-L-rhamnoside) exhibited the greatest accumulation in CO2. Additionally, quercetin 3-O-neohesperidoside and kaempferol 3-O-beta-sophoroside presented significantly greater accumulations in ZJ1 and ZJ2, whereas kaempferol-3,7-O-a-L-dirhamnoside and kaempferol 3-O-glucoside exhibited the greatest accumulations in CO1 and LF1, respectively. These results reveal complex accumulation patterns of flavan 3-ols and flavonol glycosides in the CO, LF, and ZJ cultivars. Notably, more flavan 3-ols and flavonol glycosides accumulated in the buds than in the one bud and two leaves stages of the CO, LF, and ZJ cultivars (Figure S1) and may be important factors affecting the taste of tea made from the buds or the one bud and two leaves stage of these cultivars.

### 2.4. Analysis of RNA Sequencing

**Figure 5:**
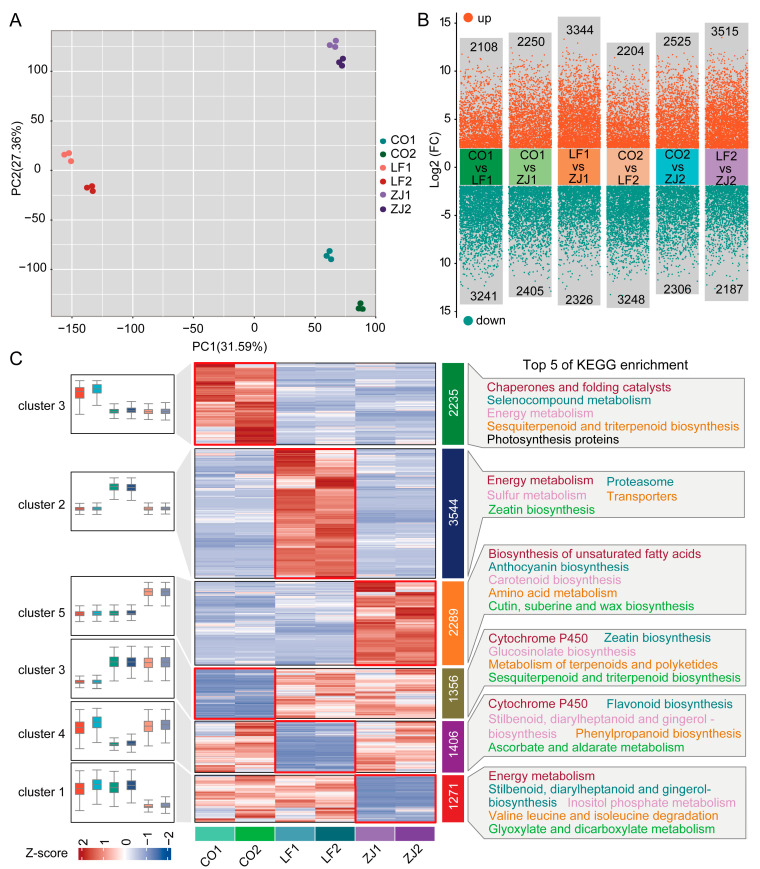
RNA-seq analysis of CO, LF, and ZJ leaves. (**A**) PCA for samples based on all genes. (**B**) Numbers of differentially expressed genes (DEGs) in different comparisons. (**C**) Expression patterns and KEGG enrichment terms of DEGs in different comparisons.

To gain insight into the important genes underlying alterations in flavonoid accumulation across different leaf samples and cultivars, an RNA-seq analysis was conducted (Table S4). A Pearson analysis indicated that there was high repeatability among the 3 biological replicate samples (Figure S2). The PCA revealed that PC1 and PC2 of the gene expression data together accounted for 58.95% of the total variance in the expressed genes and clearly distinguished six samples (Figure 5A). Nine genes were randomly selected to verify the accuracy of the transcriptome data via a qRT-PCR analysis, and the results reveal that the expression patterns of these genes detected via qRT-PCR analysis were consistent with those detected via transcriptome sequencing (Figure S3), indicating that the transcriptome sequencing results could be reliable. Subsequently, 5349, 4655, 5670, 5452, 4831, and 5702 DEGs were identified in the CO1_vs_LF1, CO1_vs_ZJ1, LF1_vs_ZJ1, CO2_vs_LF2, CO2_vs_ZJ2, and LF2_vs_ZJ2 comparison groups, respectively (Figure 5B and Table S5), indicating significant variations in the gene expression profiles of the samples of the buds and one bud with two leaves among the CO, LF, and ZJ cultivars. These DEGs were subjected to a clustering analysis, which revealed six significant distinct clusters according to their expression levels (Figure 5C). The genes in cluster 3, cluster 2, and cluster 5 presented relatively high expression levels in the CO, LF, and ZJ cultivars, respectively, whereas those in cluster 6, cluster 4, and cluster 1 presented relatively low expression levels in the CO, LF, and ZJ cultivars, respectively. A KEGG enrichment analysis revealed significantly distinct characteristics of different cluster members; specifically, ‘anthocyanin biosynthesis’ was enriched in genes whose expression was relatively high in ZJ, whereas ‘sesquiterpenoid and triterpenoid biosynthesis’ and ‘flavonoid biosynthesis’ were enriched in genes whose expression was relatively low in CO and LF, respectively, which was consistent with the metabolite results (Figure 1E).

### 2.5. Correlation Analysis of Flavonoid Levels and Related Synthetic Gene Expression

**Figure 6:**
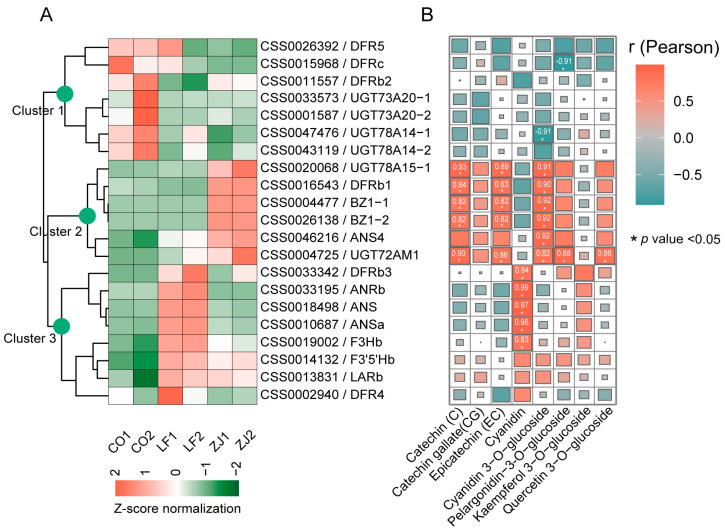
DEGs related to anthocyanidins, flavan 3-ols, and flavonol glycosides. (**A**) Clustering heatmap of DEGs related to anthocyanidins, flavan 3-ols, and flavonol glycosides. (**B**) Pearson correlation coefficients between selected DEGs and anthocyanidins, flavan 3-ols, and flavonol glycosides.

There were 21 DEGs related to the biosynthesis of anthocyanidins, flavan 3-ols, and flavonol glycosides identified in this study, including 1 *F3H*, 1 *F3′5′H*, 6 *DFR*, 1 *ANR*, 3 *ANS*, 1 *LAR*, and 8 *UGT*, respectively (Table S6). The expression of these genes was cultivar specific, and the genes clustered into three patterns, with cluster 1, cluster 2, and cluster 3 members being highly expressed in CO, ZJ, and LF, respectively (Figure 6A). An association analysis revealed that some of these DEGs were highly correlated with specific flavonoids (r > 0.8 and *p* < 0.05) and thus may contribute to their accumulation (Figure 6B and Table S7). For example, flavonol 3-O-glucosyltransferase genes (*CsUGT78A15-1*/*CSS0020068*, *CsUGT72AM1*/*CSS0004725*, *CsBZ1-1*/*CSS0004477*, and *CsBZ1-2*/*CSS0026138*) were found to be associated with cyanidin 3-O-glucoside, and *CsUGT72AM1* was also associated with pelargonidin 3-O-glucoside and quercetin 3-O-glucoside. *CsUGT78A15-1* encodes a protein highly conserved with CsUGT78A15 (Figure S4), which is a broad substrate that functionally catalyzes the formation of a series of flavonol 3-O-glucosides in tea plants; CsUGT72AM1 has been demonstrated to catalyze the formation of quercetin 3-O-glucoside and cyanidin 3-O-glucoside; and CsBZ1-1 and CsZB1-2 are referred to as anthocyanidin 3-O-glucosyltransferase (EC: 2.4.1.115) members that catalyze the formation of anthocyanidin 3-O-glucoside. In addition, our results reveal that *CsDFRb1* (*CSS0016543*) was associated with catechin (C) and epicatechin (EC), whereas *CsDFRb3* (*CSS0033342*), *CsANS* (*CSS0018498*), and *CsANSa* (*CSS0010687*) were associated with cyanidin. Together, these findings suggest the complexity of the accumulation of specific flavonoids that involve multiple genes.

### 2.6. Identification of Transcription Factors Involved in Flavonoid Biosynthesis

**Figure 7:**
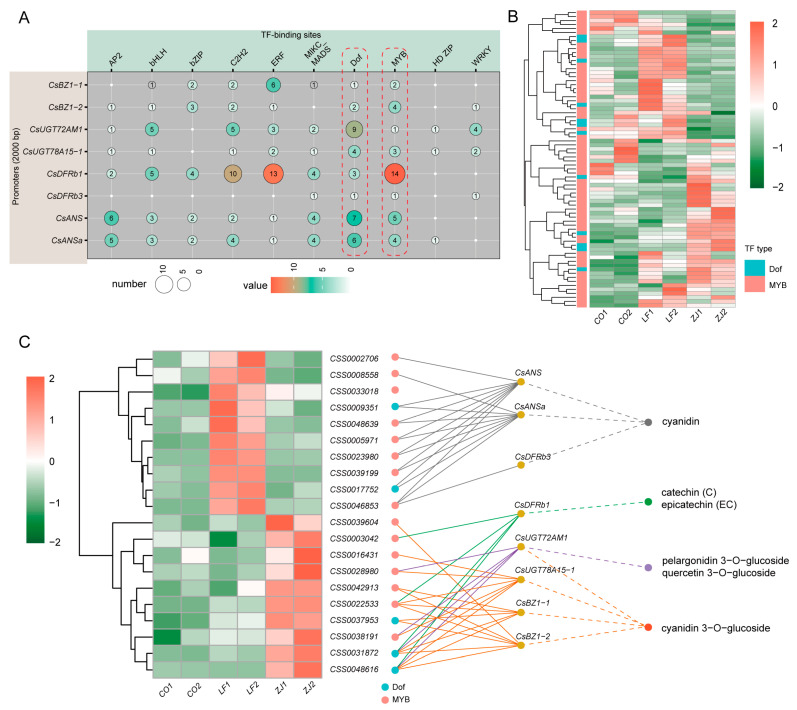
Identification of potential transcription factors (TFs) involved in biosynthesis of anthocyanidins, flavan 3-ols, and flavonol glycosides. (**A**) Cis-elements present in promoters of selected genes. (**B**) Expression of differentially expressed *MYB* and *Dof* genes. (**C**) Potential regulatory networks for anthocyanidin, flavan 3-ols, and flavonol glycoside biosynthesis in CO, LF, and ZJ.

Transcription factors (TFs) are important regulators that are involved in plant metabolism accumulation by binding to the promoters of structural genes. Therefore, to investigate the potential TFs involved in the accumulation of the specific flavonoids mentioned above, TF binding site scanning of their related structural genes was first performed. The results reveal that multiple TF binding sites were present in all of the gene promoters, whereas MYB and Dof TF binding sites were present in all of the investigated genes (Figure 7A and Table S8). A total of 62 *MYBs* and 12 *Dofs* were subsequently identified from the DEGs (Figure 7B and Table S9). Among them, 15 *MYBs* and 5 *Dofs* were highly associated with some investigated structural genes (r > 0.9 and *p* < 0.05) and were therefore considered to be involved in the accumulation of specific flavonoids (Figure 7C and Table S10). Specifically, eight *MYBs* (*CSS0002706*, *CSS0008558*, *CSS0033018*, *CSS0048639*, *CSS0005971*, *CSS0023980*, *CSS0039199*, and *CSS0046853*) and two *Dofs* (*CSS0009351* and *CSS0017752*) were predicted to be involved in cyanidin accumulation by regulating *CsDFRb3*, *CsANS*, *CsANSa*, or *CsF3Hb* expression; two *MYB* (*CSS0003042* and *CSS0022533*) and two *Dofs* (*CSS0031872* and *CSS0048616*) were predicted to be involved in catechin and epicatechin accumulation by regulating *CsDFRb1*; and six *MYBs* (*CSS0039604*, *CSS0016431*, *CSS0028980*, *CSS0042913*, *CSS0022533*, and *CSS0038191*) and three *Dofs* (*CSS0037953*, *CSS0031872*, and *CSS0048616*) were predicted to be involved in cyanidin 3-O-glucoside by regulating *CsUGT78A7815-1*, *CsBZ1-1*, or *CsBZ1-2* expression. *CSS0028980*, *CSS0038191*, *CSS0031872*, and *CSS0048616* were also predicted to be involved in the accumulation of cyanidin 3-O-glucoside, pelargonidin 3-O-glucoside, and quercetin 3-O-glucoside through regulating *CsUGT72AM1* expression. Notably, most specific structural genes were predicted to be regulated by multiple TFs, suggesting a complex regulatory network related to specific flavonoids that is mediated by multiple TFs and structural genes.

## 3. Discussion

An abundant array of tea germplasm resources, characterized by substantial diversity in the accumulation of biochemical metabolites, particularly flavonoids such as catechins (flavan-3-ols), anthocyanidins, and flavonol glycosides, exert a definitive influence on both tea processing procedures and the ultimate quality of the tea product. These flavonoids have attracted significant attention because of their health benefits and pivotal roles in imparting bitterness and astringency to tea. Additionally, anthocyanidins are crucial factors influencing the coloration of tea plant leaves. Consequently, uncovering the underlying molecular mechanisms responsible for the variations in the accumulation of these compounds among tea cultivars exhibiting distinct color phenotypes represents a promising avenue for the utilization of tea plant resources. To achieve this goal, the flavonoid levels in tea cultivars with these color characteristics were investigated, and a transcriptional regulatory network with flavonoid synthesis structural genes as its core was constructed.

Catechin, anthocyanidin, and flavonol glycoside compounds exhibit distinct variational patterns of accumulation among tea cultivars with different color phenotypes, which are attributed to alterations in the expression of related synthetase genes, including *F3′H*, *DFR*, *ANS*, *ANR*, *LAR*, *FLS*, and *UGT* [18,21,49,50]. For example, the green-leaf tea cultivar ‘Longjing 43′ presented lower expression levels of *F3′H*, *FLS*, *DFR*, and *UGT* than the purple-leaf mutant ‘Mooma 1’, resulting in decreased levels of anthocyanidin compounds, several glycosylated flavonols, and two catechin compounds [18], underscoring the complexity of the accumulation of these flavonoids in tea plants, which is orchestrated by a network of related biosynthetic genes.

In addition to the direct involvement of *ANR* and *LAR* in the biosynthesis of monomeric catechins, *DFR* and *ANS* also play synergistic roles [51,52,53]. For example, compared with green shoots, yellow mutant shoots of ‘Danzicha’ have decreased expression of *DFR* and *ANS* members, accompanied by lower catechin levels [54]. In our study, the levels of C and EC in ZJ were greater than those in CO and LF, which may be related to *DFRb1*. Moreover, other *DFR*, *ANS*, *ANR*, and *LAR* members presented relatively high expression levels in LF or CO, and these genes are primarily involved in the formation of other flavonoids not focused on in this study. UGTs play an important role in the diversity of metabolites by catalyzing the transfer of an activated sugar donor to acceptor molecules. In tea plants, several UGTs, such as CsUGT14, CsUGT15, CsUGT73A20, and CsUGT72AM1, were found to be responsible for the biosynthesis of flavonol 3-O-glucosides and anthocyanidin 3-O-glucosides [18,22,54,55]. Furthermore, BZ1 is a UGT that was suggested to catalyze the formation of anthocyanidin 3-glucoside in plants. In the present study, *CsBZ1-1*, *CsBZ1-2*, *CsUGT78A15-1*, and *CsUGT72AM1* were found to be associated with cyanidin 3-O-glucoside. Moreover, *CsUGT72AM1* was also associated with pelargonidin 3-O-glucoside and quercetin 3-O-glucoside, suggesting their synergistic role in the abundance of these compounds in ZJ. Several studies have indicated that cyanidin levels are notably greater in purple-leaf tea cultivars [21,50], whereas other reports indicate that cyanidin levels are not significantly different among yellow-leaf, green-leaf, and purple-leaf tea cultivars [56]. Interestingly, we found that the cyanidin levels in LF were greater than those in CO and ZJ because of the high expression of *CsANS*, *CsANSa*, and *CsDFRb3* in LF, which encode enzymes directly responsible for its production and upstream reactions.

Transcription factors (TFs) in plants function as critical regulators involved in the flux of flavonoids. In tea plants, several MYB members function as critical regulators of the biosynthesis of catechins and anthocyanins, affecting the expression of *DFR*, *ANS*, *ANR*, and *LAR* either directly or indirectly [38,39]. Other TFs, such as bHLH, WRKY, and HSF, also have the ability to regulate the synthesis of specific flavonoids [40,49,57]. These findings indicate a complex network of flavonoid synthesis involving multiple structural genes and transcription factors in tea plants. Our study revealed that the promoters of all of the genes responsible for the biosynthesis of specific catechins, anthocyanidins, and flavonol 3-O-glycosides contain MYB-binding *cis*-elements. Furthermore, multiple *MYB* genes were highly correlated with the expression of some of these genes, supporting the critical role of *MYB* genes in regulating flavonoid biosynthesis. Notably, despite the lack of evidence of the involvement of *Dof* genes in flavonoid synthesis in tea plants, the present study revealed that Dof-binding *cis*-elements also exist in all investigated gene promoters and that the expression of five *Dof* genes was highly correlated with the expression of some of these genes. These findings suggest the potential involvement of *Dof* genes in regulating specific flavonoids, which is worthy of further study. In addition, although the function of UGT in tea plants has been well verified, the upstream regulatory factors of *UGT* are less well studied. Our study revealed that multiple *MYB* and *Dof* genes may synergistically regulate their expression, thereby leading to variations in anthocyanidin 3-glucoside and flavonol glycoside accumulation in different tea cultivars.

## 4. Materials and Methods

### 4.1. Plant Materials

Three tea plant cultivars, ‘Mingguan’ (CO), ‘Lvyafoshou’ (LF), and ‘Zijuan’ (ZJ), which were grown in the tea garden of the Tea Research Institute, Fujian Academy of Agricultural Sciences in Fu’an, China (119° 350′ E, 27° 100′ N), were used in this study. Samples of the buds and one bud with two leaves of each cultivar were collected from at least 60 individual healthy plants and were randomly divided into 3 groups representing three biological replicates. All of the samples were immediately frozen with liquid nitrogen after being removed from the plants and stored at −80 °C for further use.

### 4.2. Metabolite Extraction and UHPLC-MS Analysis

The freeze-dried samples were crushed with a mixer mill for 30 s at 60 Hz. A 100 mg aliquot of each individual sample was precisely weighed and extracted overnight at 4 °C with 500 μL of extraction solution (methanol/water = 3:1, containing an internal standard: 0.3 mg/mL 2-Chloro-L-phenylalanine) on a shaker. The supernatant was filtered through a 0.22 μm microporous membrane, and the resulting supernatants were subsequently diluted 10 times with extraction solution and vortexed for 30 s before UHPLC-MS analysis.

UHPLC-MS analysis was carried out using a 6500+ Triple Quad LC-MS/MS System equipped with an EXIONLC UHPLC unit (AB Sciex, Framingham, MA, USA). An ACQUITY UPLC HSS T3 column (1.8 μm, 2.1 mm × 100 mm) was used for the separation of metabolite compounds. The mobile phase consisted of water containing 1% formic acid (A) and 100% acetonitrile (B), with a flow rate of 0.4 mL/min. The typical ion source parameters were set as follows: ion spray voltage, +5500 and −4500 V; curtain gas, 35 psi; temperature, 400 °C; ion source gas, 1, 60 psi; ion source gas, 2, 60 psi; and DP, ±100 V.

### 4.3. Metabolite Data Preprocessing and Analysis

SCIEX Analyst Work Station Software (version 1.6.3) was employed for multiple reaction monitoring (MRM) data acquisition and processing. The qualitative analyses of the compounds were performed by comparing the obtained mass spectra information with a self-constructed database (Metware database: MWDB). During the analysis, isotope signals, as well as repeated signals containing K^+^, Na^+^, and NH^4+^ ions, and signals from fragments of other substances with larger molecular weights, were excluded. Metabolites were quantified by calculating the area of each individual peak. A principal component analysis (PCA) of the identified metabolites was performed using the R package gmodels (version 2.19.1). On the basis of variable importance in the projection (VIP) values of the orthogonal projection to latent structures discriminant analysis (OPLS-DA) model, metabolites with VIP ≥ 1.0, a *p* value < 0.05, and|Log2 (fold change)| > 1 were defined as significantly differentially accumulated metabolites (DAMs). The R package ‘pheatmap’ (version 1.0.12) was used to generate a heatmap for visualization of DAMs.

### 4.4. RNA Sequencing and Data Analysis

Total RNA was extracted using the TRIzol reagent (Invitrogen, Carlsbad, CA, USA). The quality and integrity of the RNA from each sample were assessed by a NanoDrop spectrophotometer (Thermo Scientific, Waltham, MA, USA). The RNA library construction and sequencing were performed by Allwegene Company (Beijing, China). The paired-end 150 bp reads of each library were generated using the Illumina NovaSeq 6000 platform.

The raw sequences were transformed into clean reads by removing reads containing adapters, reads containing poly-N sequences, and low-quality reads. The clean reads were subsequently mapped to the ‘Shuchazao’ reference genome [58] by STAR. HTSeq (version 2.0) was used to count the number of reads mapped to each gene. Gene expression levels were estimated as fragments per kilobase of transcript per million fragments (FPKM). Genes with |Log2 (fold change)| >2 and an adjusted *p* value < 0.05 were considered to be differentially expressed genes (DEGs). Kyoto Encyclopedia of Genes and Genomes (KEGG) enrichment analyses were performed via TBtools software (version 2.112). The R package ‘ClusterGVis’ was employed to integrate the expression clusters and KEGG enrichment results of the DEGs.

### 4.5. Real-Time Quantitative PCR (qRT-PCR) Analysis

Nine genes were randomly selected for qRT-PCR analysis to verify the accuracy of the transcriptome results. GAPDH and PTB were used as reference genes to normalize gene expression using the 2^−ΔΔCt^ method. The specific primers used are listed in Table S1. The first-strand cDNA of each sample was prepared using the HiScript III 1st-Strand cDNA Synthesis Kit (Vazyme, Nanjing, China). qRT-PCR was performed using the ChamQ universal SYBR qPCR Master Mix (Vazyme, China) on a LightCycler 480 II platform (Roche, Mannheim, Germany).

### 4.6. Correlation Analysis and Transcriptional Regulatory Network Construction

The correlation coefficients between the selected DAM levels and the corresponding synthetic gene expressions were calculated and visualized via the R package Hmisc (version 5.1.3) and the qcorrplot function in the R package Hy4m/linkET, respectively. The expression of the Pearson correlation coefficient (PCC) between transcription factor genes and selected structural genes was calculated via the R package Hmisc (version 5.1.3). Promoters of selected genes, 2000 bp sequences upstream of the start codon, were subjected to PlantRegMap [59] to scan the TF binding sites. The transcriptional regulatory networks were constructed by correlating the PCC  >  0.9 between each TF gene and selected structural gene pairs with the corresponding TF binding sites in the promoters of the selected structural genes.

## 5. Conclusions

In this study, we conducted an integrated analysis of metabolomics and transcriptomics to investigate the networks regulating astringency and color-related flavonoids in tea cultivars with diverse leaf colors. Through this analysis, we found that EC, C, cyanidin 3-O-glucoside, pelargonidin 3-O-glucoside, and quercetin 3-O-glucoside accumulated to higher levels in the purple-leaf tea cultivar ZJ compared to the yellow-leaf tea cultivar CO and the green-leaf tea cultivar LF. However, cyanidin accumulated more abundantly in the green-leaf tea cultivar LF. These different accumulation patterns of specific flavonoids, affecting the taste and leaf color of tea plants, may be contributed by the expression of eight key structural genes controlled by 15 *MYB* genes and 5 *Dof* genes as potential regulators. Despite the established role of *MYB* genes, there is scant evidence regarding the involvement of *Dof* genes in flavonoid synthesis within tea plants, which aroused our interest and will be the focus of our future research. Overall, our findings provide insights into the development and utilization of resources from tea plants with diverse leaf colors.

## Data Availability

The original contributions presented in this study are included in the article/Supplementary Materials. Further inquiries can be directed to the corresponding authors.

## Supplementary Materials

The following supporting information can be downloaded at https://www.mdpi.com/article/10.3390/plants14040625/s1, Figure S1. Comparison of flavan 3-ols and flavonol glycoside levels between the buds and one bud with two leaves of CO, LF, and ZJ; Figure S2. Correlation analysis of average expression levels of 18 samples (n = 3); Figure S3. qRT-PCR verification of the RNA-seq results for 9 differentially expressed genes (DEGs) in CO, LF, and ZJ samples. The data are shown as the mean ± standard deviations (SD) (n = 3); Figure S4. Sequence alignment of CsUGT78A15 and CsUGT78A15-1; Table S1. Primers used in this study; Table S2. All 863 metabolites were altered; Table S3. DAMs in different comparison groups; Table S4. Summary of RNA-seq results of 18 samples; Table S5. Information on DEGs in different comparison groups; Table S6. Information on flavonoid-related DEGs; Table S7. Pearson correlation coefficients between structural genes and specific flavonoids; Table S8. Information on TF binding sites of investigated structural genes; Table S9. Information on differentially expressed MYB and Dof genes; Table S10. Pearson correlation coefficient between each TF gene and selected structural gene pairs.
