# Supplementary material for: Revealing the Molecular Regulatory Mechanism of Flavonoid Accumulation in Tender Leaves of Tea Plants by Transcriptomic and Metabolomic Analyses
Source: Plants (Basel). 2025 Feb 19;14(4):625. doi: 10.3390/plants14040625 (PMC11859652; doi:10.3390/plants14040625)
Supplement: Supplementary file 1 [file plants-14-00625-s001.zip › Supplementary figures.pdf]

SUPPORTING INFORMATION

Revealing the Molecular Regulatory Mechanism of Flavonoid Accumulation in Tender Leaves of Tea Plants by Transcriptomic and Metabolomic Analyses

Ruiyang Shan [1,†], Yongheng Zhang [2,†], Xiaomei You [1], Xiangrui Kong [1], Yazhen Zhang [1], Xinlei Li [1], Lu Wang [2], Xinchao Wang [2,*] and Changsong Chen [1,*]

Supporting Figures

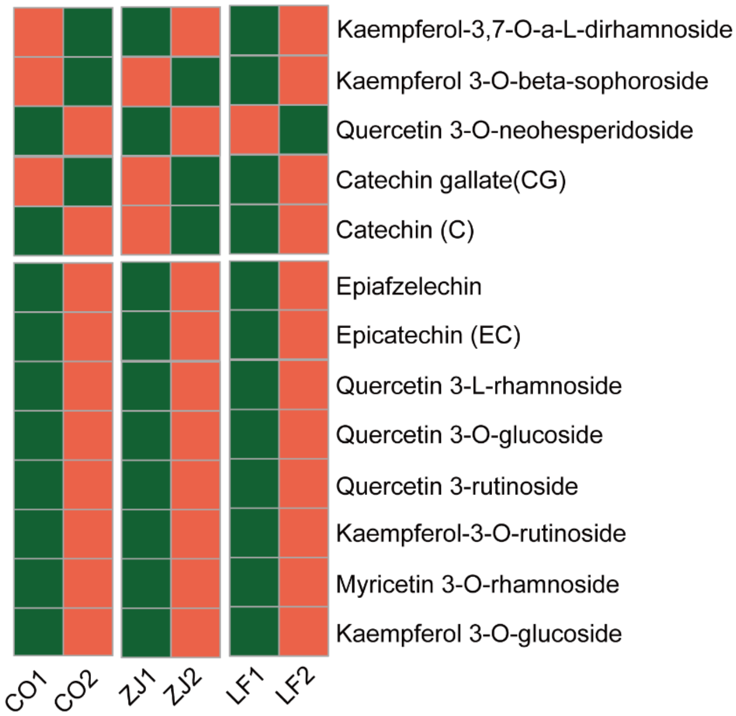

Figure S1. Comparison of flavan 3-ols and flavonol glycoside levels between the buds and one bud with two leaves of CO, LF, and ZJ.

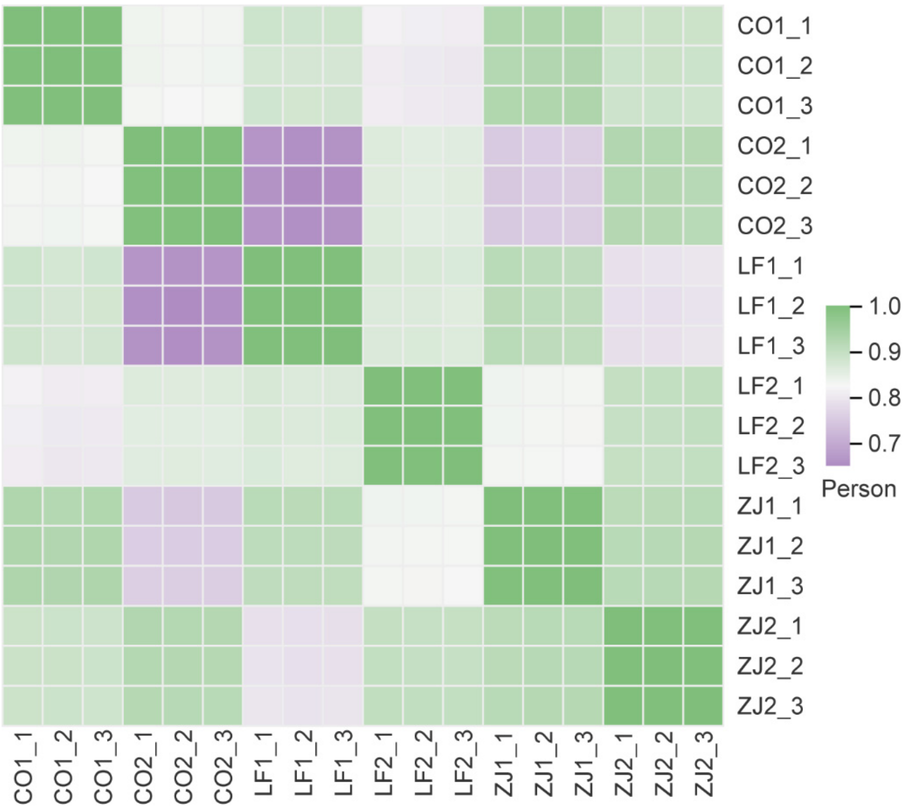

Figure S2. Correlation analysis of the average expression levels of 18 samples (n=3).

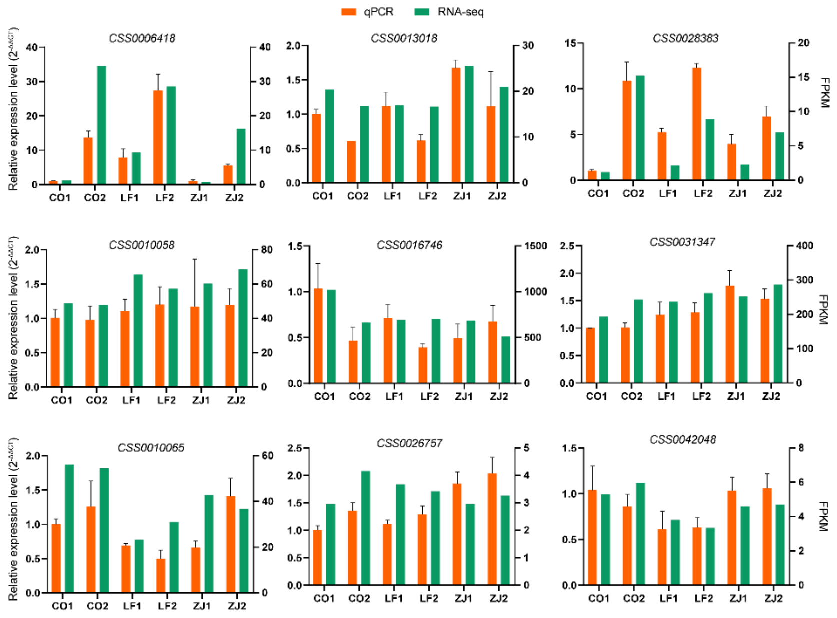

Figure S3. qRT-PCR verification of the RNA-seq results for 9 differentially expressed genes (DEGs) in CO, LF, and ZJ samples. Data are mean ± standard deviations (SD) (n=3).

```
CsUGT78A15   MSTMVTNSSLPERHIAVLAFPFASHAGLTLGLIRRLATASVDVTFSFYSTAKSIQYLLSSSPIPDNIKPCHVLDEYVFSENMGDIELFLKVGKECFKRAMKATEEETGRRISCVMADAFV   120
CSS0020068   ........................................................................................................................     0

CsUGT78A15   WFSGDMAEEMRVPWVPLWTSGACSLSTHCYTDLIRETVGIHGIAGRENEILKFVPGFPELRLGDLPSGILFGNLESAFSIMLHKMGQTLPKATAVLINSFEELDPEINKNLNSKFLKFLN   240
CSS0020068   ....................................MDANAGIAGRENEILKFVPGFPELRLGDLPSGILFGNLESAFSIMQHKMGQALPKATAVLINSFEELDPEINKDLNSKFLKFLN    84

CsUGT78A15   VGPFNLTSPPRLSNSDDYGCISWLDKRKPTSVAYIGFGTVAKPTPDELVALAEALEASTTPFLWSMEDKLKEQLPEGFLTRTSEQGKIVAWAPQVQILAHISTGVFITHCGWNSVLESIA   360
CSS0020068   VGPFNLTSPPRLSNSDDYGCISWLDKRKPTSAAYIGFRTVAKPTPDELVALAEALEASTTPFLWSMEDKLKEHLPEGILTRTSEQGKIVAWAPQVQILAHISTGVFITHCGWNSVLESIA   204

CsUGT78A15   AGVPMIGRPFFGDHHINTWMVENVWKIGVRVEGGVFTKIGTMHALELVLSHERGRKLKEQIGHFKELALKAVGPKGSSSQNFCTLLEVVTSLNL.......   454
CSS0020068   AGVPIIGRPFFGDHHINTWMLENVWKIGVRVEGGVFTKSGTMDALEIVLSHERGRKLKEQIGCFKELALMAVGPKGSSSQNFNTLLAMVTSLNLGKCMEGL   305
```

Figure S4. Sequence alignment of CsUGT78A15 and CsUGT78A15-1.
